# Predictive Potential of Circulating Ube2h mRNA as an E2 Ubiquitin-Conjugating Enzyme for Diagnosis or Treatment of Alzheimer’s Disease

**DOI:** 10.3390/ijms21093398

**Published:** 2020-05-11

**Authors:** Key-Hwan Lim, Jae-Yeol Joo

**Affiliations:** Neurodegenerative Diseases Research Group, Korea Brain Research Institute, Daegu 41062, Korea; khlim@kbri.re.kr

**Keywords:** Alzheimer’s disease, E2 ubiquitin conjugating enzyme, Ube2h, ubiquitin

## Abstract

Neurodegenerative disorders are caused by neuronal cell death, miscommunications between synapse, and abnormal accumulations of proteins in the brain. Alzheimer’s disease (AD) is one of the age-related disorders, which are the most common degenerative disorders today, and strongly affects memory consolidation and cognitive function in the brain. Amyloid-β and tau proteins are triggers for AD pathogenesis, and usually used as AD candidate biomarkers in the clinical research. Especially, clinical exam, brain imaging and molecular biological methods are being used to diagnosis for AD. Genome-wide association study (GWAS) is a new biomedical method, and its use contributes to understanding many human diseases, including brain diseases. Here, we identified ubiquitin conjugating enzyme E2 (Ube2) gene expression in neurons through GWAS. The subfamilies of Ube2’s genetic expression and inborn errors affect the ubiquitin proteasome system (UPS), leading to protein degradation in the brain. We found that only Ube2h mRNA transcription was significantly increased in the blood from AD, however we did not find any change of Ube2 subfamily genes’ expression in the blood and brain tissue. These data may provide information for diagnosis or clinical approach, and suggest that cell-free circulating Ube2h mRNA is a novel potential biomarker for AD.

## 1. Introduction

Alzheimer’s disease (AD) is a genetically inherited disease, and is also caused by multifactorial factors, which has become a very well-appreciated neurodegenerative disorder model, recently [1]. Clinically, hallmarks of AD are mutations of the early-onset of amyloid precursors, Presenilin-1 (PS1) and Presenilin-2 (PS2), as integral membrane proteins [2]. However, various mutations of late-onset hallmarks, which have no inheritance of AD, are also reported in many clinical cases [1]. Although identification of apolipoprotein E (APOE) was added as a new major component of AD, and is increased in AD, specific populations, such as in Africa, have no mutation of APOE genes [3]. This surprising genetical finding can be explained by the high-throughput genomic approach, known as Genome-wide association study (GWAS). Because retinal and intestinal systems can also share various genes between AD and Parkinson’s disease (PD), identification of new specific players which have intracellular toxicity and quantification through GWAS may be a very useful system for the application of the clinical diagnosis of AD [4,5]. Given that AD and PD as neurodegenerative disorders have much cross-talk in the gene expression network, the finding of a new specific maker will suggest the way for diagnosis or treatment of AD. Despite the substantial progress with GWAS, which has revealed the mutation or expression of target genes in various diseases including AD, there remains considerable questions as to whether the genetic code can affect the level of translated protein, and these advances would be approachable in the clinical trial [6].

Most proteins undergo posttranslational modifications (PTMs) to regulate maintaining of homeostasis [7]. Ubiquitination is a PTM that enables attachment of the ubiquitin to target proteins through E1 (activating), E2 (conjugating), and E3 (ligating) enzyme cascade [7]. Ubiquitination is initiated by activation of ubiquitin through E1s, in an ATP-dependent reaction, and transferred to E2s. Finally, E2s and E3s make a specific complex with its targeted substrates, and carboxyl terminus of ubiquitin is conjugated to the ε-amino group of lysine residues on the target protein [7]. This reaction also serves to assemble the ubiquitin chains through seven lysine residues, such as K6, K11, K27, K29, K33, K48, and K63, on ubiquitin [7]. Early biochemical studies have shown that K11 and K48 ubiquitin chains lead to 26S proteasomal degradation, and K63 is associated with various intracellular functions, such as autophagy, DNA repairand controlling of cell cycle [8]. In addition, structural studies revealed that ubiquitin chains make many types of branched form on target proteins, and suggested that they may have a central role in ubiquitin-dependent pathways [9].

Since less than 40 E2s are encoded in mammals, it has been regarded that the E2s are orchestrated with E3s through making a ubiquitin transferring complex [10]. Ubiquitin-conjugation (UBC) domain was found in all E2s, and this domain is required for its own activation and binding of E3s [11]. Structural analysis suggests that extension of the amino- or carboxyl-terminus on each E2s showed distinct functions in their enzymatic activity [11]. *Ube2h* is identified in yeast (named as *Ubc8*) and human placenta, and belongs to the family of E2s which are structurally and functionally conserved [12,13,14]. In humans, expression of UBE2H is found in skeletal and cardiac muscles, and controlled by tumor necrosis factor-alpha/nuclear factor kappa B (TNF-α/NF-κB) as a cytokine signaling [15]. Furthermore, mutations of *Ube2h* were found in the patients who have amyotrophic lateral sclerosis (ALS), a motor neuron disease in old ages [16,17]. This clinical data indicated that *Ube2h* may be associated with neurodegenerative disorders [16,17]. Moreover, recent genetic studies reported that *Ube2h* is a meaningful gene in brain development and human brain diseases, such as autistic disorder [16,18]. These results indicated that *Ube2h* is highly polymorphic, and mutations affect neurodegenerative disorder. This phenotype is shaped by genotype–phenotype relationships.

Blood contains various type of RNAs, such as messenger RNA (mRNA), micro RNA (miRNA) and other non-coding RNA (ncRNA). Theses circulating RNAs play a crucial role in disease and are important potential biomarkers [19,20]. We here identified *Ube2h* mRNA, which is an AD specific cell-free circulating mRNA using high-throughput total RNA-sequencing (RNA-seq) from blood. Moreover, we present a quantitative analysis of E2 enzyme expression, that reveals *Ube2h*-dependent regulation in AD model. We validate the protein level in tissues and cells on functional levels, to explain the molecular linkage of transcription to translation consequences on *Ube2h*, and suggest the possibility of circulating *Ube2h* mRNA as a target of AD for clinical diagnosis and treatment.

## 2. Results

### 2.1. Characterization of Ube2 Subfamily Genes Expression in the Primary Cortical Neurons from RNA-Seq Data Base

**Figure 1:**
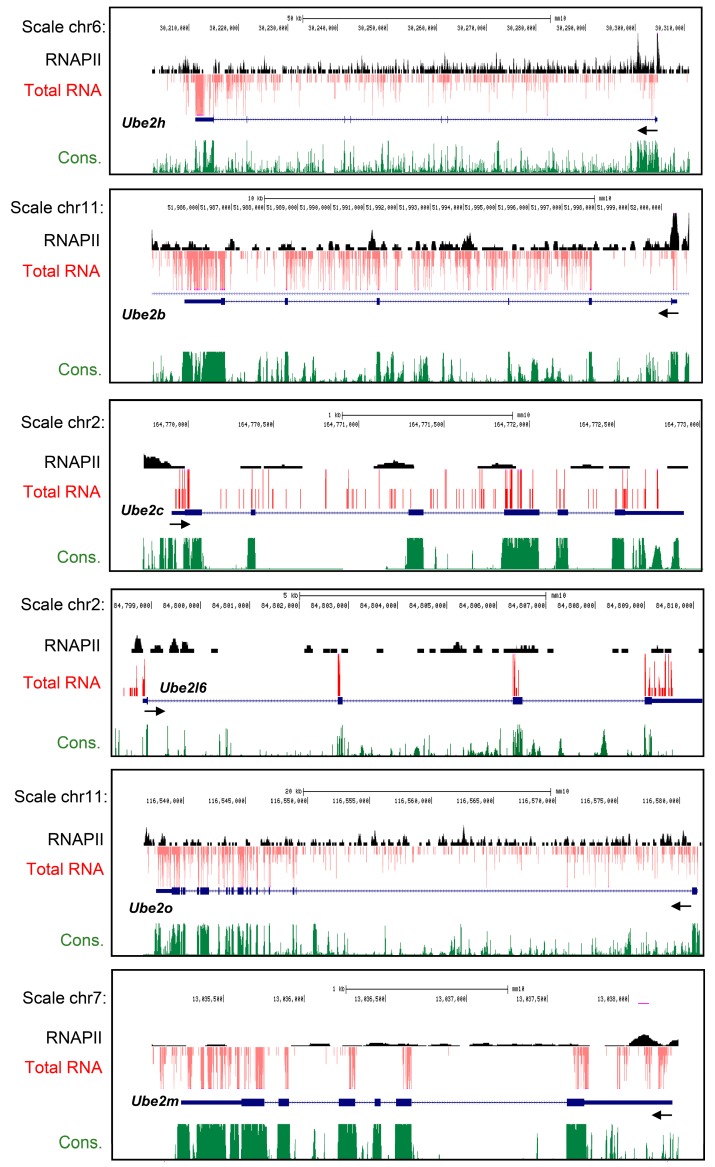
Genome-wide gene expression profile for ubiquitin conjugating enzyme E2 (*Ube2*) subfamily in the cortical neurons at days in vitro (DIV) 6. (**A**–**F**) Genome browser view of the *Ube2* subfamilies genes genomic locus, with total RNA-seq data expression of *Ube2h*, *Ube2l6*, *Ube2b*, *Ube2c*, *Ube2o*, and *Ube2m* mRNA in cortical neurons. Those *Ube2* subfamily genes are well expressed in the cultured E16.5 cortical neurons.

We first applied the mRNA expression and fragments per kilobase of transcript per million (FPKM) value, and then mapped each gene distribution in the neurons. To confirm whether *Ube2* subfamilies were well expressed in the neurons, we reanalyzed the published total RNA-seq data. *Ube2* subfamilies’ gene expression profiles were adapted from the data generated by Kim et al. (2010) [21]. In silico mining of total RNA-seq analysis revealed that the *Ube2h*, *Ube2l6*, *Ube2b*, *Ube2c*, *Ube2o* and *Ube2m* genes were highly expressed in the primary cortical neurons (Figure 1 A–F). Taken together, these results suggest that at least six *Ube2* subfamily gene expressions were well conserved in the cortical neurons.

### 2.2. Ube2h mRNA is Abundantly Expressed in the Blood from AD

**Figure 2:**
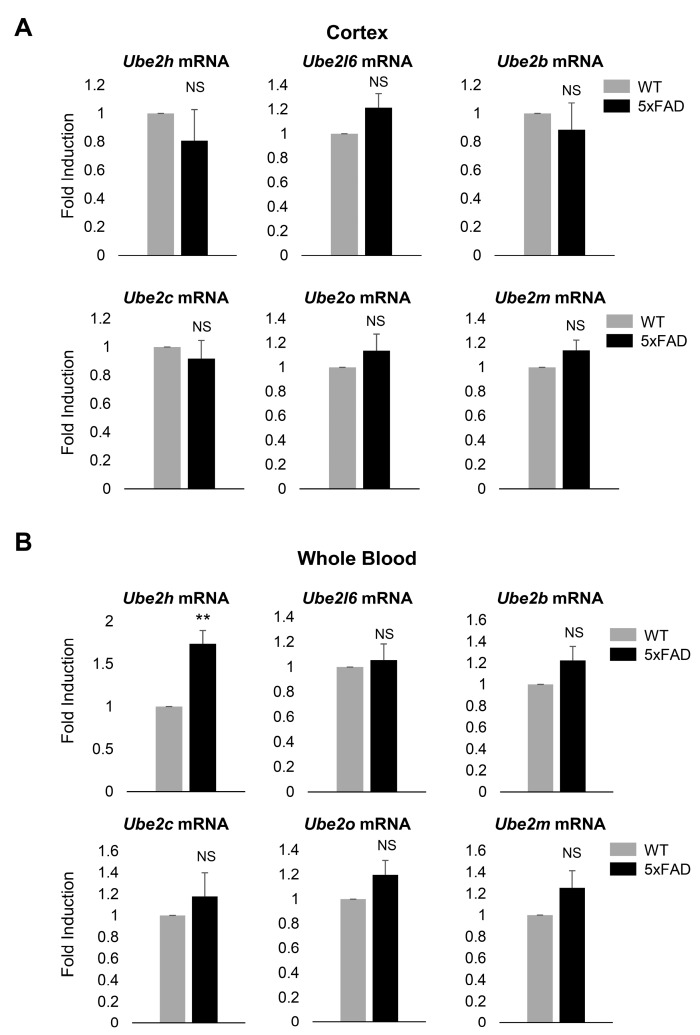
*Ube2* subfamilies’ mRNA transcription profile from AD model mouse cortex and whole blood. (**A**) RT-qPCR analysis of *Ube2h*, *Ube2l6*, *Ube2b*, *Ube2c*, *Ube2o* and *Ube2m* mRNA expression in mouse cortex from WT and AD model. Data are mean ± standard error of the mean (s.e.m.) from *n* = 3 mice per group; unpaired two-tailed *t*-test. (**B**) RT-qPCR analysis of *Ube2h*, *Ube2l6*, *Ube2b*, *Ube2c*, *Ube2o* and *Ube2m* mRNA expression in mouse whole blood from WT and AD model. Data are mean ± s.e.m. from *n* = 3 mice per group; unpaired two-tailed *t*-test.

Recent studies have suggested that the ubiquitin-proteasome system (UPS) was dysfunctional in brain diseases such as schizophrenia [22]. *Ube2k* enzyme and mRNA transcription levels are increased in blood and brain tissue from post-mortem schizophrenia patients. Six *Ube2* subfamily genes expression were confirmed by total RNA-seq data from cortical neurons. To determine whether the expression of *Ube2* subfamily genes was AD specific, we performed quantitative reverse transcription PCR (RT-qPCR) from whole cortex and blood. We designed six different primers to amplify the specific region of each *Ube2* subfamily genes. The expression levels of the *Ube2l6*, *Ube2b*, *Ube2c*, *Ube2o* and *Ube2m* mRNA did not show significant changes in both wild type (WT) and 5xFAD in the cortex and whole blood (Figure 2A). In the contrast to *Ube2l6*, *Ube2b*, *Ube2c*, *Ube2o* and *Ube2m* mRNA, *Ube2h* mRNA expression levels were only elevated in 5xFAD in whole blood. However, there was no significant change of the *Ube2h* mRNA expression level in the cortex (Figure 2B). Taken together, these results suggest that the *Ube2h* mRNA was highly expressed in blood at 5xFAD.

### 2.3. Accurate Prediction of Gene Expression Change through Total RNA-Seq for AD from Whole Blood

**Figure 3:**
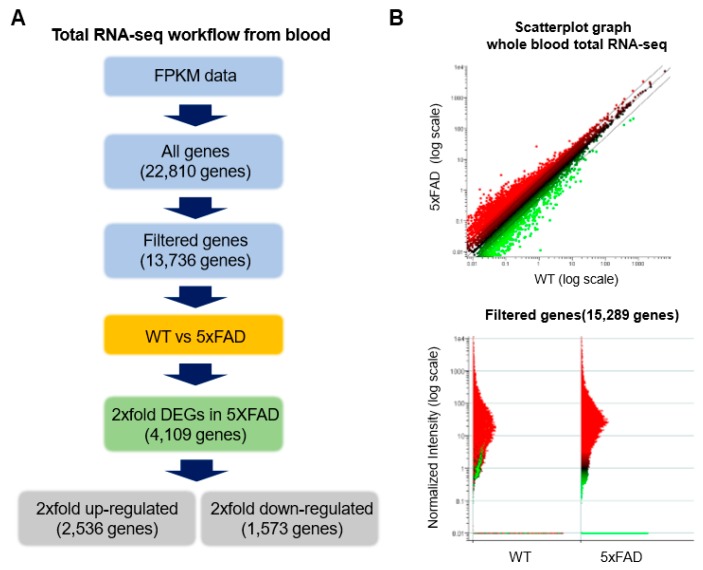
Total RNA-seq workflow from mouse whole blood. (**A**) Total RNA-seq analysis. Single and paired-end reads calculated from NGS sequencing. Mapping with preprocessing from Ras sequencing data, and then filtered with differently expressed genes from WT and 5xFAD. 4,109 genes are two-fold change of DEG between WT and 5xFAD. (**B**) Assessing normalization of total RNA-seq using scatterplot matrices from WT and AD model. X-axis indicates gene expression of WT, Y-axis indicates gene expression of AD.

**Figure 4:**
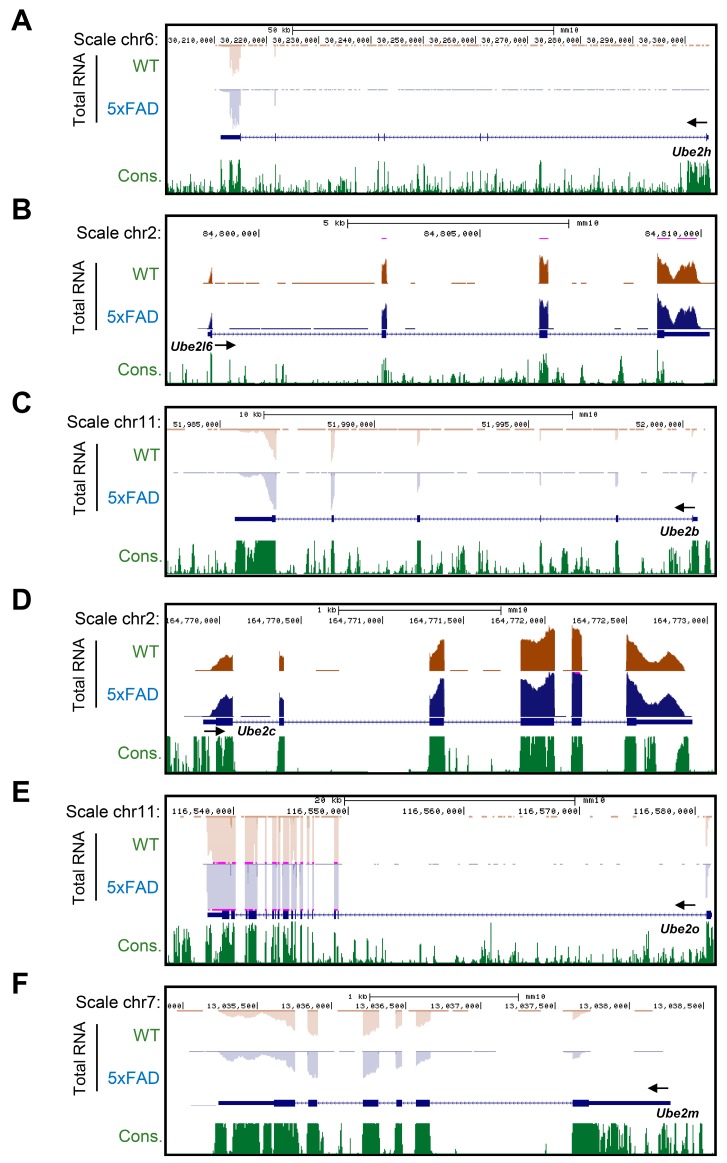
Gene expression change profile through total RNA-seq for AD from whole blood. (**A**–**F**) Genome browser view of the genomic locus for *Ube2* subfamily genes, with total RNA-seq data expression of *Ube2h*, *Ube2l6*, *Ube2b*, *Ube2c*, *Ube2o*, and *Ube2m* mRNA in whole blood from WT and AD model. Expression of *Ube2h* mRNA was increased in AD model. On the other hand, there was no change of *Ube2l6*, *Ube2b*, *Ube2c*, *Ube2o*, and *Ube2m* gene expression level between WT and AD model.

RNA-seq is an approach to gene expression profiling that uses high-throughput sequencing technology, developed around the last decade. The aim of total RNA-seq is to quantify the changing gene expression levels of each gene’s transcription in the cell, tissue or blood, and so on. Annotate gene expression profiling method is based on Agilent GeneSpring analysis, and the image was generated by FASTQ illumine package bcl2faseq. Each gene’s FPKM value was calculated through Cufflinks package, and we then collected 22,810 genes from whole blood. For further validation, we sorted 4109 genes, which were two-fold change DEG (differentially expressed genes), between WT and 5xFAD. To identify AD-dependent specific gene expression, we subsequently performed the Gene Ontology (GO) functional classification protocol. 2536 and 1573 genes were up- or down-regulated in whole blood (Supplementary Tables S1 and S2). We therefore considered that around 4109 genes were dynamically expressed in the whole blood, and these genes might play an important role for AD pathology (Figure 3A,B; Supplementary Figure S1). We next explored whether *Ube2* subfamily genes are AD-specifically expressed in cortex and whole blood. First, we monitored the mRNA level of the *Ube2* subfamily genes *Ube2h*, *Ube2l6*, *Ube2b*, *Ube2c*, *Ube2o* and *Ube2m* by RT-qPCR (Figure 2), and then we normalized the total RNA-seq data based on six types of *Ube2* subfamily genes expression in WT whole blood. We found *Ube2h* mRNA’s expression level was increased in comparison to WT (Figure 4A). By contrast, there was no change in *Ube2l6*, *Ube2b*, *Ube2c*, *Ube2o* and *Ube2m* mRNA expression level between WT and AD in whole blood (Figure 4B–F). Interestingly, this total RNA-seq data was correlated with RT-qPCR (Figure 2B).

### 2.4. Biochemical Characterization of UBE2H on AD in Cortex

**Figure 5:**
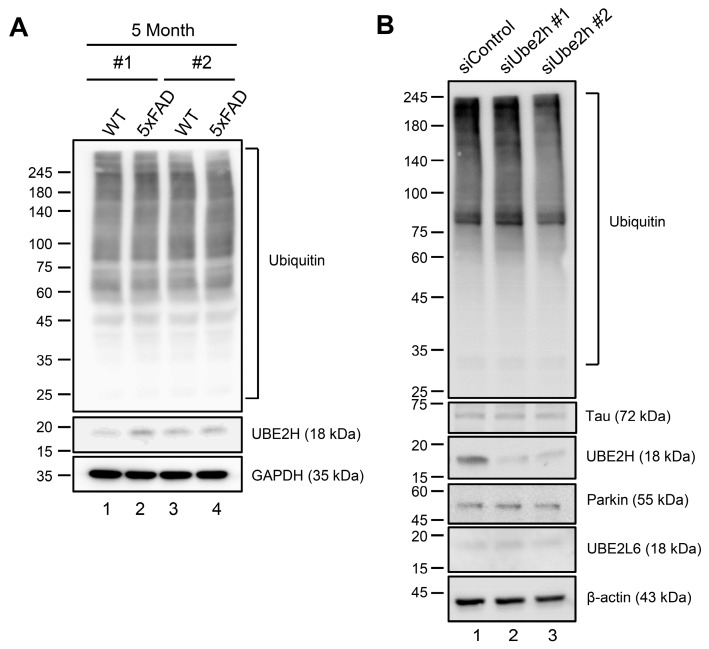
UBE2H was up-regulated in the whole cortex of AD (**A**) UBE2H was increased in AD model. Cortex tissues were obtained from 5XFAD mouse, and lysates were analyzed by immunoblotting with the indicated antibodies. (**B**) Knockdown of *Ube2h* did not affect a UBE2 family and AD related protein expression. HEK 293T cells transfected with indicated siRNAs for 48 h and harvested. Endogenous proteins, including UBE2H levels, were analyzed by immunoblotting.

We speculate that abundant expression of *Ube2h* may be associated with an accumulation of proteolysis-related protein, and lead to neurodegenerative disorder. Therefore, we next evaluated whether *Ube2h* is enriched for protein expression in AD, given the main role of this protein in regulation of the ubiquitin-dependent process. We first tested the expression levels of UBE2H and ubiquitin in both WT and 5xFAD. As we expected, UBE2H was increased in AD compared to WT (Figure 5A, lane 2 and 4). However, as we did not find any changes in the level of endogenous ubiquitinated proteins caused by increments of UBE2H (Figure 5A), we reasoned that ubiquitinated proteins existed most abundantly in the tissues. To investigate the effects on AD-related proteins and the UBE2 family by the level of expression for UBE2H, we depleted UBE2H using siRNA (Figure 5B). Depletion of UBE2H led to significantly decreased endogenous expression for UBE2H, but did not affect the expression of Tau and Parkin as neurodegeneration disorder proteins, or UBE2L6 as a UBE2 family (Figure 5B, lane 2 and 3). Collectively, our results implicate that the UBE2H is increased in AD, thereby it is contributing to the AD development gradually.

### 2.5. Analysis of Ube2 Subfamilies’ Gene Expression Profiles in AD Patients from Peripheral Blood Mononuclear Cells (PBMCs) Microarray Dataset

**Figure 6:**
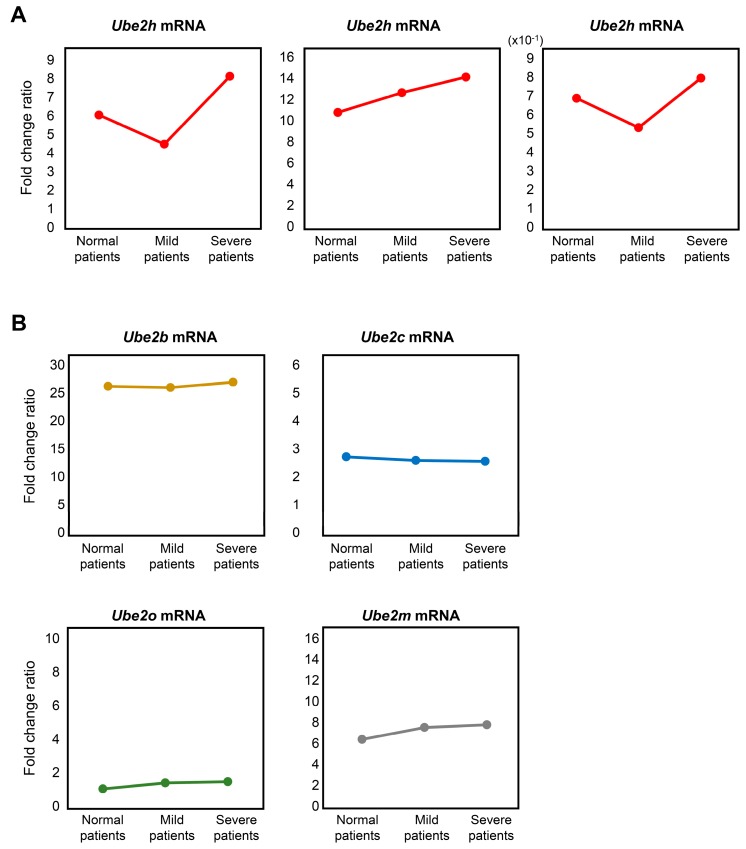
Gene expression profile of *Ube2* subfamilies from AD patients in PBMC. (**A**) *Ube2h* mRNA expression in AD patients through PBMCs microarray data set. (**B**) Differential *Ube2* subfamilies’ mRNA expression in PBMCs of AD patients.

Having found a change of gene expression for *Ube2* subfamilies in AD in whole blood, we next analyzed the human *Ube2* subfamilies’ gene expression profiles in AD patients through PBMC’s microarray data set [23]. In order to confirm the target *Ube2* subfamilies’ gene expression profiles, we compared the severe AD patients’ PBMCs gene expression with normal and mild patients. Gene difference analysis revealed that there were five differential *Ube2* subfamilies’ mRNA in patients’ PBMCs. To correlate the *Ube2h* gene expression result in 5xFAD whole blood, we then selectively compared the level of fold change ratio from three different probe sets in AD patients’ PBMCs. Interestingly, we found that *Ube2h* mRNA expression levels are elevated in severe AD patients (Figure 6A). On the other hand, *Ube2b*, *Ube2c*, *Ube2o* and *Ube2m* mRNA expression levels are not changed between normal and severe AD patients (Figure 6B). These results strongly suggest that the *Ube2h* gene is AD-specifically elevated in both 5xFAD and human AD patients.

## 3. Discussion

The AD pathology is mainly characterized in the cerebral cortex and hippocampus, and extensively connected with other neurodegenerative disorders such as PD [24,25]. Traditionally, clinical examinations, including blood tests, brain structural imaging and family history, have been used to determine the symptoms for AD. In addition, the diagnosis of AD is dependent on the disease progression and biomarker profiles. However, the onset and progression of AD in patients is clinically variable, and biomarker assessment and the molecular mechanisms remain poorly understood [26]. Most of all, the limitations of these approaches are due to the lack of knowledge about AD. Therefore, a better understanding of the molecular pathogenesis, with identification of simple and certain biomarkers, is required in improving the treatment of AD.

GWAS is the most powerful approach to genetic variation and gene function discovery of various human diseases [27,28,29,30]. This technique has revealed that eukaryotic gene expression is extensively regulated by long- and short-ncRNA [31,32,33]. We previously characterized neuronal enhancer activation, and suggested an understanding of the physiological and functional mechanism of gene expression in relation to various neural activity-dependent plasticity, with GWAS in cortical neurons [34,35]. In this study, we firstly sorted *Ube2* subfamily genes through GWAS in cortex and whole blood, then identified cell-free circulating *Ube2h* mRNA as a new specific marker for diagnosis of AD. Although mRNA of *Ube2* subfamilies were highly transcribed in both cortex and primary cortical neurons, interestingly, only *Ube2h* mRNA was significantly increased in whole blood from AD models and AD patients’ PBMCs (Figure 2, Figure 3 and Figure 6). Even though we did not show any information concerning why the blood included high *Ube2h* mRNA in the AD model, we suggest that stable biofluids, which included specific circulating miRNAs or mRNAs, could be changed upon some damage to various organs [36,37]. Gene expression profiling in blood is an important method in clinical approach and molecular study. The human blood circulation system plays an important role in the cell to cell network, such as transport of nutrients, and reception or exchange of external information. Cell-free RNAs were identified in serum from malignant melanoma patients around two decades ago [38]. Previous studies have shown that the cell-free transcript was strongly correlated with many human diseases, and specific circulating RNAs were suggested as potential biomarkers for diagnosis of various human diseases. Indeed, the use of circulating mRNA from patient-derived blood has been essential for diagnostic tests of various human diseases [39,40,41,42,43,44,45,46]. Recently, the blood samples were employed in vascular pathology, leading to a suggestion for the landscape of blood-based biomarkers in AD [47]. Therefore, our AD model-derived analysis, including bloods with the respective circular *Ube2* mRNA, could be used to investigate AD pathological phenotypes. However, using a gene-based assay to quantify the frequency of target gene expression often showed unexpected features of the protein translation [48]. Although the expression of UBE2H is greatly increased (Figure 5A), similar to other *Ube2* genes, *Ube2h* mRNA did not show a difference of transcription in the AD cortex (Figure 2B). It is not clear how E2 family genes allow this phenomenon. Moreover, the function of *Ube2h* in AD is not known so far. One model poses that so-called extracellular vesicles (EV) are required to concentrate *Ube2h* from blood to cortex, where the presence of this EV somehow activates the expression of *Ube2h* [49].

Part of the molecular mechanism of AD is the accumulation of amyloid beta (Aβ) and Tau, associated with mitochondrial dysfunction and mitophagy [50]. In the previous study, *Ube2* subfamilies were found to be a co-factor for Parkin-dependent mitophagy. Most notably, knockdown of *Ube2* subfamilies did not lead to degradation of Parkin proteins that make reductions of mitochondrial polyubiquitination [51]. Interestingly, depletion of *Ube2h* lacked the expression of polyubiquitination, and did not affect the levels of Tau and Parkin (Figure 5B), suggesting that UBE2H also may contribute to the ubiquitin-dependent system in the cortex, excluding Parkin and Tau as the well-known AD biomarkers. The exact mechanism by which UBE2H regulates AD-related ubiquitination is currently unknown. UBE2H may directly accumulate the enzymatic activity of E3s or its binding proteins. The UBC domain on UBE2 subfamilies may mediate the interaction with unknown AD-related factors, and increments of this interaction could lead to ubiquitination processes in AD. Suppression of *Ube2h* by siRNAs supports this possibility (Figure 5).

Transcription and translation processes are closely linked, resulting in expression of proteins to regulate homeostasis in organs. The translation and expression of UBE2H, a known E2s for the ubiquitin-dependent system in the posttranslational modification, promotes target protein ubiquitination [51]. This is believed as E2s result from the UBE2H-mediated ubiquitin-conjugating enzymatic activity [17]. Likewise, we observed that depletion of UBE2H decreased intracellular polyubiquitination. Although *Ube2* subfamilies have integrated in AD, the function of these proteins remains largely unexplored. Given the role of the ubiquitin-conjugating enzyme, possibly a strong association of AD, the existence of the *Ube2h* gene in various tissue, including blood, is expected to be a new potential biomarker for prediction and diagnosis of AD without brain biopsy or cerebrospinal fluid (CSF) detection. Indeed, specific expression of circulating *Ube2h* mRNA in blood could be essential for AD patients, and could contribute to rapid diagnosis or prognosis with high specificity (Figure 6). The growing list of E2s characterized by powerful genetic system, as revealed in this study, greatly improve the isolation or identification of the potential proteins which have fundamental cellular functions in the treatment of AD.

## 4. Materials and Methods

### 4.1. Animals

5xFAD transgenic mice were used as models of Alzheimer’s disease. 5xFAD mice were obtained from the Jackson Laboratory. All animal experiments performed in this study were reviewed and approved by the IACUC committee at Korea Brain Research Institute (IACUC-20-00018).

### 4.2. RNA-Seq

Total RNA was isolated from mouse whole blood using conventional TRIzol method (Invitrogen). To profile gene expression from mouse whole blood, mouse blood (0.8 mL) was used for purification of the total RNA. Libraries were prepared using TruSeq Stranded Total RNA LT Sample Prep Kit (Human Mouse Rat, Illumina Inc., San Diego, CA, USA), according to the Illumina’s protocol. Briefly, fragmentation buffer was added to mRNA for short fragments first. Taking these short fragments as templates, oligo dT-primer was used to synthesize the first-strand cDNA. The second-strand cDNA was synthesized using buffer, dNTPs (containing dUTP instead of dTTP), RNaseH and DNA polymerase I, respectively. Double-stranded cDNA was purified with QIAQuick PCR extraction kit (Qiagen, Hilden, Germany) and resolved with elution buffer. Following the synthesis of second strand, end repair, addition of single A base, and adaptor ligation, cDNAs were connected with sequencing adaptors. The concentration of each library was measured by real-time PCR. Agilent 2100 Bioanalyzer (Santa Clara, CA, USA) was used for profiling the distribution of insert size. Constructed libraries were sequenced by Illumina HiSeq™ 4000, based on the manufacturer’s instructions which were sequenced for 100 cycles. The HiSeq Control Software HCS (v3.3) with RTA (v2.7.3) (Illumina Inc.), was used to provide the management and execution of the HiSeq™ 4000 experiment runs.

### 4.3. Sequencing Data Analysis

Images generated by HiSeq™ 4000 were converted into nucleotide sequences by base calling, and stored in FASTQ format utilizing Illumina package bcl2faseq (v2.16.0.10). By filtering the dirty reads, which contained adaptors, unknown or low Phred quality scored bases, from raw reads, clean reads were generated. Clean reads were mapped to reference UCSC hg19 genome and gene sequences using Tophat2 (v2.1.0). Mismatches of no more than 5 bases were allowed in the alignment. To annotate gene expression, fragments per kb per million reads (FPKM) values of each gene were calculated using Cufflinks package (v2.2.1). To analyze differentially expressed genes (DEGs), fold-change analysis was performed using Agilent GeneSpring (v7.3). For the expression pattern analysis, hierarchical clustering analysis was performed using Agilent GeneSpring (v7.3). Functional enrichment analysis was done using Gene Ontology (GO) functional classification system (www.geneontology.org) and DAVID website (http://david.ncifcrf.gov). Raw reads and data are accessible in the Gene Expression Omnibus (GSE 147792)

### 4.4. Microarray Data

Microarray datasets GSE18309 were downloaded from EMBL-ENA website (www.ebi.ac.uk/ena). The GSE18309 datasets were derived from human PBMCs, and their microarray experiments were performed using Affymetrix Human Genome U133 Plus 2.0 Array (Affymetrix, Santa Clara, CA, USA).

### 4.5. Data Analysis

The obtained raw data were analyzed through a series of python commands, and visualized using Agilent GeneSpring 7.3. Briefly, 9 raw datasets were normalized by dividing each gene’s intensity values by each sample’s 50% percentile value. Secondly, all genes in samples of the same group (control, mid, AD) were averaged, and finally, to calculate the differentially expressed genes, the averaged value in mid and AD group were normalized by that of the control group. The genes with fold change ratio >2 or <0.5 were considered as DEGs.

### 4.6. RT-qPCR

Total RNA was extracted from mouse cortex or whole blood using the TRIzol method. cDNA synthesis was done using High-Capacity reverse transcription kit (Applied Biosystems) according to the manufacturer protocols. Primers employed were: *Ube2h* coding forward, 5′-CCATAGATCCTCTCAATGGTG-3′, reverse, 5′-CTCTTCCGTTGCGTACTTCT-3′; *Ube2l6* coding forward, 5′-CTCAAAGCCTTCCAAGTGCG-3′, reverse, 5′-CAGGCACACCAGACCATCCT-3′; *Ube2b* coding forward, 5′-GAATGGAAGTTAAGATGGAAGA-3′, reverse, 5′-GCTGATGGTAGCATATGTTTAG-3′; *Ube2o* coding forward, 5′-CCAATCCAGGTACCTAGGAG-3′, reverse, 5′-CCCCACTTTTGCTACCTCTC-3′; *Ube2m* coding forward, 5′-CATTTAAGAAATACCTGCAAGAA-3′, reverse, 5′-ATCAGGGATCTTGGCTGGAG-3′; *Ube2c* coding forward, 5′-GAGGATGTTGAGGCAGACGT-3′, reverse, 5′-GGGACAGGGTTACCCACAT-3′. Statistical significance was evaluated by an unpaired two-tailed *t*-test.

### 4.7. Cell Culture, siRNA Transfection and Immunoblotting

Human embryonic kidney (HEK) 293T cells were cultured in Dulbecco’s Modified Eagles Medium (DMEM, Hyclone) supplemented with 1% penicillin/streptomycin (P/S) and 10% fetal bovine serum (Hyclone). Predesigned siRNA duplexes were purchased from Bioneer, and 10 nM siRNA transfection was carried out using RNAiMAX (Invitrogen, Waltham, MA, USA). Cells and tissues were lysed with N-PER™ and T-PER™ lysis buffer (Thermo Scientific, Waltham, MA, USA) supplemented with protease inhibitor cocktail (Roche, Hoffmann-La Roche AG, Basel, Switzerland), respectively. Lysates were resolved by SDS-PAGE gels and transferred onto nitrocellulose (NC) membranes (Merck Millipore, Burlington, MA, USA), and then antibodies were detected using the D-Plus ECL Femto System (Dongin Bio, Seoul, Korea) and the ImageQuant LAS4000 (GE Healthcare, Chicago, IL, USA). Antibodies used for immunoblotting included the following: anti-Ubiquitin (P4D1, Santa Cruz, Santa Cruz Biotechnology, Dallas, TX, USA), anti-UBE2H (18-Z, Santa Cruz), anti-Parkin (PRK8, Santa Cruz), anti-GAPDH (0411, Santa Cruz), anti-β-actin (C4, Santa Cruz), anti-Tau (Abcam, Cambridge, UK), and anti-UBE2L6 (MyBioSource, San Diego, CA, USA).

## Supplementary Materials

The following are available online at https://www.mdpi.com/1422-0067/21/9/3398/s1, Figure S1. Dynamic gene expression from whole blood RNA-seq data in AD model. Table S1. GO analysis result from WT and 5xFAD in whole blood. Table S1. GO analysis result from WT and 5xFAD in whole blood. Table S2. Profile of upregulated or downregulated genes in 5xFAD whole blood through Kyoto Encyclopedia of Gene and Genomes (KEGG) pathway analysis.
